# A Case of Third Dentition, Etc.

**Published:** 1852-07

**Authors:** J. Harris

**Affiliations:** Salem, Ohio


### Original Communications.

For the Dental Register of the West.

### A CASE OF THIRD DENTITION, ACCOMPANIED WITH A MODEL OF THE TEETH AND GUMS.

James Holliday, a colored man about five feet ten inches high, well proportioned, intelligent countenance, and hair white, was one hundred and five years of age when the accompanying model of his mouth was taken. His health was apparently good, he could hear remarkably well, and in reply to my inquiry respecting his sight, he took up a Hymn Book of the usual type, and read with great facility, without glasses.

As the notes which I took of the case at the time, have accidentally been mislaid, it will be out of my power to particularize as to dates, &c.

Our subject was born a slave in the State of Maryland, where he resided for more than half a century. He then removed to New Jersey, and after remaining there for several years, he came to this neighborhood, where he died a few years ago.

About thirty-six years previous to the interview alluded to above, his second set of teeth which were sound, began to loosen and drop out, one at a time, commencing at the incisors and progressing backwards, at intervals of nearly equal lengths of time, till there was but one remaining in his mouth, which as you will observe by the model, appears to be the second molaris on the right side of the upper jaw, and readily distinguishable from those of the third set by its greater length. The tooth immediately anterior to the one last described, had made its appearance within the last year, and judging from the looseness of the remaining survivor of the second set, it probably came out within a year or so afterwards. There was no apparent disease of the alveolus, and the removal of the second set with the other phenomena connected with the third dentition, was attended with but little uneasiness.

The texture of the third set of teeth seemed to be unusually brittle, the attrition consequent upon their use in mastication having reduced their length, especially those of the upper set, almost to a level with the adjacent gums.

It appeared in the case just described, as if nature had pursued her ordinary course with our subject, until he had arrived at about the usual age allotted to our race, when, it seems as if a renovation of certain parts of his system had been brought about; that of his sight to much perfection, while in the case of his teeth, a predominance of the calcarious principles over that of the gelatinous, gave them an unusual degree of brittleness; they are also, smaller than the second set.

J. HARRIS.

Salem, Ohio, May, 1852.

---

The description of this singular case of third dentition, is so accurate that it needs no comment. For the articulating model we would return Dr. Harris our sincere thanks.

In Good's Noosology, I think we find one or two cases of a similar character. The idea is suggested, if the man had lived a few years longer, would the remaining tooth have been displaced? We think it would.—Ed.

